# Coordinated alteration of mRNA-microRNA transcriptomes associated with exosomes and fatty acid metabolism in adipose tissue and skeletal muscle in grazing cattle

**DOI:** 10.5713/ajas.19.0682

**Published:** 2019-12-24

**Authors:** Susumu Muroya, Hideki Ogasawara, Kana Nohara, Mika Oe, Koichi Ojima, Masayuki Hojito

**Affiliations:** 1 Animal Products Research Division, NARO Institute of Livestock and Grassland Science (NILGS), Tsukuba, Ibaraki 300-1207, Japan; 2 Field Science Center, School of Veterinary Medicine, Kitasato University, Yakumo, Hokkaido 049-3121, Japan

**Keywords:** Exosome, Grazing Cattle, Lipid Metabolism, microRNA, Organ Crosstalk, Subcutaneous Fat

## Abstract

**Objective:**

On the hypothesis that grazing of cattle prompts organs to secrete or internalize circulating microRNAs (c-miRNAs) in parallel with changes in energy metabolism, we aimed to clarify biological events in adipose, skeletal muscle, and liver tissues in grazing Japanese Shorthorn (JSH) steers by a transcriptomic approach.

**Methods:**

The subcutaneous fat (SCF), biceps femoris muscle (BFM), and liver in JSH steers after three months of grazing or housing were analyzed using microarray and quantitative polymerase chain reaction (qPCR), followed by gene ontology (GO) and functional annotation analyses.

**Results:**

The results of transcriptomics indicated that SCF was highly responsive to grazing compared to BFM and liver tissues. The ‘Exosome’, ‘Carbohydrate metabolism’ and ‘Lipid metabolism’ were extracted as the relevant GO terms in SCF and BFM, and/or liver from the >1.5-fold-altered mRNAs in grazing steers. The qPCR analyses showed a trend of upregulated gene expression related to exosome secretion and internalization (charged multivesicular body protein 4A, vacuolar protein sorting-associated protein 4B, vesicle associated membrane protein 7, caveolin 1) in the BFM and SCF, as well as upregulation of lipolysis-associated mRNAs (carnitine palmitoyltransferase 1A, hormone-sensitive lipase, perilipin 1, adipose triglyceride lipase, fatty acid binding protein 4) and most of the microRNAs (miRNAs) in SCF. Moreover, gene expression related to fatty acid uptake and inter-organ signaling (solute carrier family 27 member 4 and angiopoietin-like 4) was upregulated in BFM, suggesting activation of SCF-BFM organ crosstalk for energy metabolism. Meanwhile, expression of plasma exosomal miR-16a, miR-19b, miR-21-5p, and miR-142-5p was reduced. According to bioinformatic analyses, the c-miRNA target genes are associated with the terms ‘Endosome’, ‘Caveola’, ‘Endocytosis’, ‘Carbohydrate metabolism’, and with pathways related to environmental information processing and the endocrine system.

**Conclusion:**

Exosome and fatty acid metabolism-related gene expression was altered in SCF of grazing cattle, which could be regulated by miRNA such as miR-142-5p. These changes occurred coordinately in both the SCF and BFM, suggesting involvement of exosome in the SCF-BFM organ crosstalk to modulate energy metabolism.

## INTRODUCTION

MiRNAs are non-coding small RNAs that play a role in the suppression of translation and induction of mRNA degradation in a variety of cellular biological events, which occurs via interaction with the 3′-untranslated region of the target mRNAs [1]. The functional miRNAs are originally transcribed from their genes, followed by processing into the mature ~22-nucleotide-long RNAs, via pre-miRNAs. Some miRNAs have been shown to be loaded into a subgroup of extracellular vesicles (EVs) with a diameter in the range of 30 to 100 nm, i.e., exosomes, and thereby are secreted into a variety of body fluids, such as blood, saliva, breast milk, and urea [2]. In the past decade, the key regulatory roles played by miRNAs in solid tissues as well as in bodily fluids during exercise have been revealed [3].

We have shown that miRNAs in skeletal muscle and in the circulating exosomes of cattle are affected by both grazing and feeding. The levels of exosomal miR-19b [4] and miR-142-5p in plasma were lowered, whereas that of miR-208b in the *biceps femoris* muscle (BFM) was elevated [5] in grazing Japanese Shorthorn (JSH) steers as compared to housed steers. Pasture-fed grazing Japanese Black steers showed higher miR-10b levels and lower levels of other miRNAs such as miR-19a, miR-29b, miR-98, miR-142-5p, and miR-425-5p in the plasma exosomal fraction, compared to that in samples from grain-fed steers [6]. In healthy human subjects, acute and endurance exercise is also known to alter serum and/or plasma levels of skeletal muscle-associated miRNAs, suggesting that levels of circulating miRNAs (c-miRNA) are affected by exercise and physiological conditions [3].

In cattle, grazing can be considered as physical exercise. In humans, moderate-intensity exercise induces changes in energy metabolism, resulting in fatty acid generation and release from adipose tissues into the circulation [7]. With even a short period of inactivity, decreased insulin sensitivity, reduced postprandial clearance, loss of muscle mass, and visceral fat accumulation are observed [8], indicating that physical activity in exercise is indeed associated with the maintenance of energy homeostasis. During exercise, energy homeostasis is mediated by signal molecules between liver and adipose tissue and the skeletal muscles [9]. Moreover, EVs and their contents, microRNAs (miRNAs), have been identified as potential mediators of organ crosstalk [10,11]. It is hypothesized that grazing affects intra-organ metabolism and inter-organ crosstalk in cattle. Since adipose tissue plays a critical role not only in the storage of fatty acids as energy substrates, but also in the secretion of energy homeostasis mediators [12], crosstalk between adipose tissue and other organs could be potentially important for energy homeostasis throughout the whole body, impacting gene expression related to energy metabolism in secretory organs.

To date, numerous studies have shown that the content and distribution of miRNAs in circulating exosomes depend on the originating cell type and reflect its physiological and pathological states [13]. Changes in c-miRNAs are of interest as a sign of physiological stress and disease, indicating the potential use of c-miRNAs as biomarkers for monitoring disease status and as therapeutic probes. miRNA-conveying exosomes secreted from skeletal muscle cells [14] and adipocytes [15] potentially play an important role in organ crosstalk, similarly to myokines and adipokines such as insulin-like growth factor 1, myostatin, adiponectin, and fatty acid binding protein 4 [FABP4]) [8,16,17]. Recently, adipose tissue has emerged as a major exosome-secretory tissue, suggesting the principal role of adipose-derived exosomes in organ crosstalk in mice [17]. Such observations have suggested that miRNA-loading exosomes in cattle are secreted from and/or internalized into secretory organs in response to changes in feeding, behavior, and environmental conditions.

Thus, the aim of the present study was to gain a better understanding not only of the potential crosstalk between secretory organs, but also of the involvement of miRNA-loading exosomes in the organ crosstalk that occurs in grazing cattle. To address this, we investigated the transcriptomic changes in subcutaneous fat (SCF), in the BFM, and in the liver under the influence of grazing in JSH cattle, with particular focus on changes in energy metabolism-related gene expression. We also discuss how grazing alters gene expression in the secretary organs via potential regulation by miRNAs.

## MATERIALS AND METHODS

### Animals

The animals were cared for as outlined in the Guide for the Care and Use of Experimental Animals established by the Animal Care Committee of the School of Veterinary Medicine at Kitasato University. The animal experimentation protocol was approved by the President of Kitasato University via judgment by the Institutional Animal Care and Use Committee of Kitasato University (Approval no. 18-048). Eight JSH steers were raised solely on grass from the pastures of the Yakumo Experimental Farm of Kitasato University. The steers were divided into two groups at the age of 17 to 20 months (mo) and weighing 398 to 530 kg: 4 cattle in the housed group and 4 in the grazing group. The grazing cattle were pasture-fed starting in late April, whereas the housed cattle were fed in a free-stall barn with grass harvested every morning in the same period, so that the average total digestible nutrients (TDN) and crude protein (CP) contents did not differ between groups. No animals in either group were fed any grain throughout the feeding period.

### Sample preparation

Blood samples were drawn from the jugular vein of each animal and the plasma was prepared as 0.1% ethylenediaminetetraacetic acid, followed by storage at −80°C until use. Blood samples were collected at 0, 1, 2, and 3 mo after the start of the grazing period. Samples in the middle sections of SCF, BFM, and liver were also taken by biopsy at 3 mo. The biopsy procedure was as follows: the animal was locally anesthetized by an intramuscular injection of 0.06 mg/kg of xylazine (Bayer, Tokyo, Japan) and a subcutaneous injection of 400 mg of lidocaine (AstraZeneca, Osaka, Japan); subsequently, a 3- to 5-cm incision was made in the skin overlying the target tissues. All samples were immediately frozen in liquid nitrogen and stored at −80°C until use.

### Plasma sample processing

We previously succeeded in preparing miRNA-enriched EVs as exosomes [4,6]. In the present study, we used the same method to prepare miRNA-containing EVs from plasma. In brief, 10 mL of the plasma sample were mixed with 20 mL of phosphate-buffered saline (PBS) and the mixture was centrifuged at 1,200 g, 4°C for 20 min. The supernatant was centrifuged at 12,000 g, 4°C for 45 min and further centrifuged at 110,000 g, 4°C for 120 min. The precipitation was suspended in PBS and centrifuged at 110,000 g, 4°C for 70 min. The final precipitation was resuspended in PBS, stored at 4°C for a few days, and then processed for RNA preparation.

### Measurement of plasma non-esterified fatty acid

Plasma non-esterified fatty acid (NEFA) concentration was determined using a NEFA C-Test Wako kit (Fujifilm-Wako, Tokyo, Japan) and an iMARK Microplate reader (Bio-Rad, Hercules, CA, USA) according to the respective manufacturer’s protocol.

### RNA preparation

Total RNA including miRNA was extracted from SCF, BFM, liver, or plasma exosome samples using the mirVana microRNA isolation kit (Thermo Fisher Scientific, Waltham, MA, USA) for microarray analysis according to the manufacturer’s protocols. The quantity and quality of the RNA were determined using an Agilent Bioanalyzer 2100 with an RNA 6000 Pico Kit (Agilent Technologies, Santa Clara, CA, USA). The total RNAs of the solid tested tissues were also employed for the polymerase chain reaction (PCR) analysis.

### Microarray analysis

The miRNA samples of SCF, BFM, or liver for 4 steers of each feeding treatment (grazing or housed) were pooled and mixed together, and then were applied to a custom microarray, SurePrint G3 8×60K (Agilent, USA) corresponding to miRBase ver. 19. Likewise, the pooled SCF, BFM, or liver mRNA samples were also applied to a Bovine (v2) Gene Expression 4×44K Microarray (Agilent, USA). The signals of the hybridized probes were detected using an Agilent Microarray Scanner (Agilent, USA), and the miRNA and mRNA results were normalized to the 90th and 75th percentile, respectively, using GeneSpring GX (Agilent, USA).

### cDNA synthesis

For miRNA samples, the cDNA was synthesized from 250 ng of total RNA for SCF, BFM, and liver samples or 9 μL of the final product of RNA preparation for exosome samples, using the miScript II RT kit (Qiagen, Tokyo, Japan) at 37°C for 60 min, and then the enzyme was inactivated at 95°C for 5 min. For mRNA samples, the cDNAs for SCF, BFM, and liver samples were synthesized from 1,000 ng of total RNA using the ReverTra Ace quantitative polymerase chain reaction (qPCR) RT kit (Toyobo, Osaka, Japan).

### Quantitative polymerase chain reaction analysis

The qPCR for mRNAs was performed using the QuantiTect SYBR Green PCR Kit (Qiagen, Japan) with primers listed in Supplemental Table 1, employing ribosomal protein L7 (*RPL7*) as an internal control. The miRNA qPCR was performed using the miScript SYBR Green PCR kit (Qiagen, Japan) in combination with the miScript Primer Assay (Qiagen, Japan). The tested target miRNAs were shown as follows: adipogenesis-associated miR-17-92a cluster (miR-18a, miR-19b, miR-20a, miR-92a), miR-21-5p, miR-103, miR-128, miR-140, miR-143, miR-145, miR-148a, miR-223 [18], miR-24-3p, miR-27b, miR-30a-5p, miR-30d, miR-107, fat accumulation-associated miR-34 [19], adipogenesis/obesity/lipolysis-associated miR-10b [19,20], multi-functional miR-142-5p [21], obesity-associated miR-185, miR-199a-5p [20], and other miRNAs (miR-23b-3p, miR-25, miR-29b, miR-28, miR-126-3p, miR-376b, miR-411a) for all the tissues, myogenesis or skeletal muscle maintenance-associated miRNAs (miR-1, miR-133b, miR-206, miR-208b, miR-486 [22]) only for BFM, and liver-specific miR-122 [23] only for liver. Differences in the expression ratios of the target miRNA/miR-15 for plasma samples and of the target miRNA/RNA U6A small nuclear for SCF, BFM, and liver samples were compared between groups. Both qPCRs were conducted according to the manufacturer’s protocol (Qiagen, Japan), using the CFX96 thermal cycler (Bio-Rad, USA). Melting curve analysis was used to confirm the specificity of the amplification reactions.

### Prediction of miRNA target genes and functional annotation of genes of interest

The miRNA target genes were predicted using the TargetScan database (Release 6.2, http://www.targetscan.org/). To classify the target genes according to functional annotation, both gene ontology (GO) and pathway analysis were performed on the target genes of miRNA differentially expressed in grazing and housed cattle, based on the qRT-PCR results. In this study, Database for Annotation, Visualization and Integrated Discovery (DAVID) bioinformatic resources (version 6.7, http://david.abcc.ncifcrf.gov) were applied to the potential target genes with the setting of *bos taurus* as the background species; this was done to enrich GO terms and characteristics of the Kyoto encyclopedia of genes and genomes (KEGG) pathway terms defined by KEGG (http://www.genome.jp/kegg/) for the respective miRNA-mediated biological process. Extraction of the terms was considered significant when the Benjamini p-value was <0.05.

### Statistical analysis

The expression data are shown as means±standard errors and were compared between the feeding treatments by Student’s *t*-test. The effects of feeding condition were considered significant if p<0.05. Significance levels of p≤0.10 were considered a statistical trend.

## RESULTS

### High responsiveness of SCF to grazing shown by mRNA and miRNA transcriptome analyses

To investigate the response of bovine tissues to grazing, we conducted mRNA and miRNA transcriptomic analyses of the SCF, BFM, and liver at 3 mo of grazing. The total gene numbers of detected mRNA expression in the SCF, BFM, and liver were 13,989, 10,611, 12,492 in the grazing group, and 13,761, 11,633, 12,356 in the housed group, respectively. Of these, a total of 2,079, 839, and 1,653 genes in SCF, BFM, and liver of grazing cattle showed >1.5-fold expression compared to the respective tissues in the housed cattle, while 2,086, 2,940, and 1,040 genes in SCF, BFM, and liver of the grazing group showed a <0.67-fold level of expression compared to that of the housed (Table 1). The results of mRNA transcriptomic analyses indicated that SCF was a highly responsive tissue, whereas the liver was relatively stable among the tissue types tested.

On the other hand, the miRNA transcriptomic analyses showed that a total of 221, 176, and 144 miRNAs were detected in SCF, BFM, and the liver of grazing cattle, and 251, 166, and 145 miRNAs were detected in those of the housed cattle, respectively. Of these, 46, 10, 7 miRNAs in the SCF, BFM, and liver of the grazing group showed a >1.5-fold greater level of expression than in the respective samples from the housed group, while 10 and 2 miRNAs in SCF and BFM, respectively, of the grazing group showed <0.67-fold expression levels than in the housed group (Table 1). In all of the tested tissues, the highest number of changed miRNAs was observed in the SCF and the lowest was in the liver; these findings mirrored the trend observed in the mRNA transcriptomic results.

### Gene expression changes associated with extracellular exosomes as well as with lipid and carbohydrate metabolism in SCF upon grazing

Using the mRNA transcriptomic profiles, we then conducted functional annotation analyses of those genes for which expression levels exceeded a 1.5-fold increase or decrease in the tissues of grazing cattle compared to housed cattle; these functional analyses were performed to predict biological events induced in the respective tissues. In SCF, GO terms including ‘Extracellular space’ (cellular component), signaling pathways of glucagon and PPAR (KEGG pathway) were extracted in DAVID from the upregulated genes, while terms including ‘Extracellular exosome’ (cellular component), ‘Butanoate metabolism’, ‘Synthesis and degradation of ketone bodies’, ‘Glutathione metabolism’, ‘PI3K-Akt signaling pathway’, and ‘ECM receptor interaction’ (KEGG pathway) were extracted from the downregulated genes (Benjamini p<0.05, Table 2). In BFM, the GO terms including ‘Basement membrane’, ‘Extracellular exosome’, ‘Caveola’ (cellular component), ‘Propanoate metabolism’, ‘PPAR signaling pathway’, ‘Fatty acid degradation and metabolism’ (KEGG pathway) were extracted from the genes with a >1.5-fold upregulation in grazing cattle (Benjamini p<0.05, Table 2). From the downregulated genes, GO terms such as ‘Inositol phosphate metabolism’, signaling pathways of adipocytokine, NF-Kb, phosphatidylinositol and PI3K-Akt (KEGG pathway) were extracted (Benjamini p< 0.05, Table 2).

Moreover, the GO term ‘Extracellular exosome’ was also extracted from the downregulated genes in liver, as well as ‘Extracellular space’ and ‘Basement membrane’ (Table 2). These GO analysis results revealed that the activity of grazing induced molecular biological events that were associated with exosomes, with the metabolism of carbohydrates and lipids, and with extracellular matrix remodeling in the SCF, BFM, and liver of cattle.

### Exosome-related mRNA expression altered in SCF and BFM of grazing cattle

**Figure 1:**
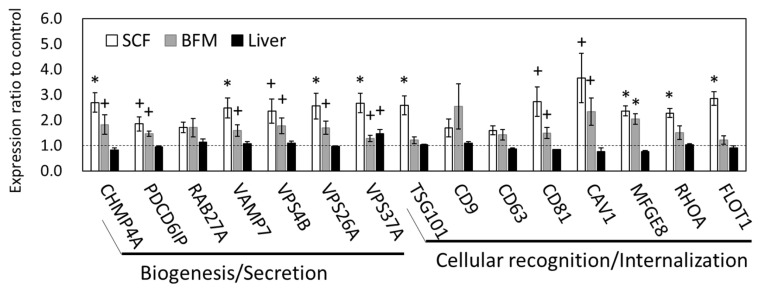
Gene expression related to exosome biogenesis, secretion, and internalization in SCF, BFM, and liver grazing cattle. The ratios of the normalized gene expression of grazing cattle to housed cattle are shown in columns (level of housed cattle is indicated with dashed line). RPL7 was used as the internal control. SCF and BFM indicate subcutaneous fat and *biceps femoris* muscle, respectively. *RPL7*, ribosomal protein L7. Error bars indicate standard error. * and + indicate differences between grazing and housed at p<0.05 and p<0.10, respectively.

The SCF showed the most prominent, grazing-induced changes in mRNA and miRNA transcriptomes among the three tissue types examined; we therefore focused on changes in SCF transcriptomes for further analysis. As the GO results indicated, there were changes in gene expression in the exosomal structural component. We then conducted qPCR analysis of the gene expression levels related to the processes of exosome biogenesis, secretion, and internalization. The qPCR results showed that the expression levels of charged multivesicular body protein 4A (*CHMP4A*), vesicle associated membrane protein 7 (*VAMP7*), vacuolar protein sorting-associated protein (VPS) 26A (*VPS26A*), *VPS37A*; tumor susceptibility 101 (*TSG101*) (p<0.05), programmed cell death 6 interacting protein (*PDCD6IP*, encoding ALIX) (p = 0.078), and *VPS4B* (p = 0.071) were upregulated in the SCF of grazing cattle, indicating that the genes related to exosome biogenesis and secretion appear to be influenced at the transcriptional level by grazing (Figure 1). Moreover, the expression of genes related to exosome internalization, namely, milk fat globule-EGF factor 8 protein (*MFGE8*), ras homolog family member A (*RHOA*), and flotillin 1 (*FLOT1*) (p<0.05), *CD81* (p = 0.052), and caveolin 1 (*CAV1*) (p = 0.053), was also upregulated in the SCF of grazing group. Meanwhile, the expression levels of *RAB27A* (a member of the small GTPase Rab family), *CD9*, and *CD63* did not significantly differ between the two cattle-feeding groups (p>0.10). Similar to that of SCF, the expression of *CHAM4A*, *PDCD6IP*, *VAMP7*, *VPS4B*, *VPS26A*, *VPS37A*, *CD81*, *CAV1* (p<0.10), and *MFGE8* (p = 0.011) was also upregulated in the BFM of the grazing cattle. As compared to the corresponding expression levels in the SCF and BFM, the gene expression levels related to exosome secretion and internalization were much less affected in the liver (Figure 1).

### Expression of plasma exosomal miRNAs altered by grazing

**Figure 2:**
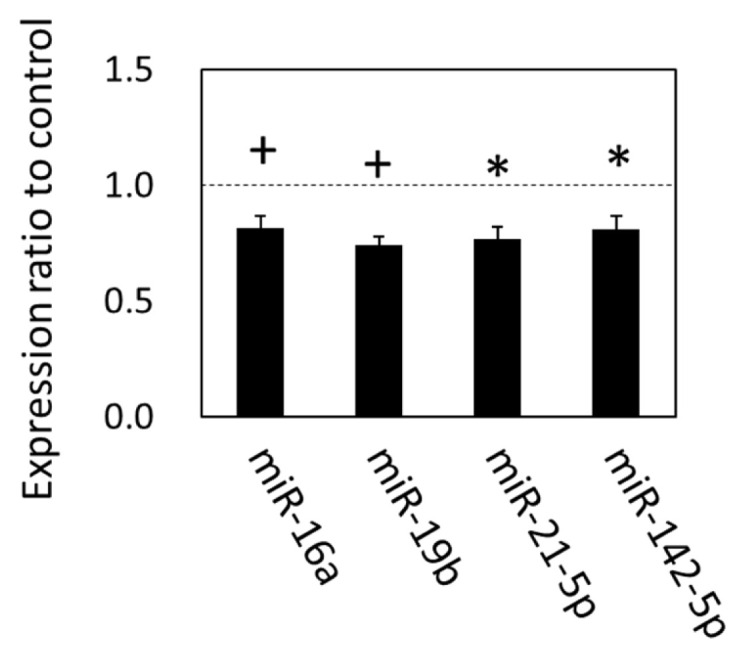
MicroRNA expression in plasma exosomes of grazing cattle. The ratios of the normalized gene expression of grazing cattle to housed cattle are shown in columns (level of housed cattle is indicated with dashed line). miR-15a was used as the internal control. Error bars indicate standard error. * and + indicate differences between grazing and housed cattle at p<0.05 and p<0.10, respectively.

The alteration of gene expression related to the exosomal components, biogenesis, and internalization suggested that the exosomal miRNAs in circulation were also altered in grazing cattle. We therefore analyzed the expression of plasma exosomal miRNAs with qPCR. The results showed that expression levels of miR-21-5p, miR-142-5p (p<0.05), miR-16a (p = 0.055), and miR-19b (p = 0.080), were downregulated in the grazing cattle (Figure 2), while the other miRNAs examined were not altered by grazing, which indicates that grazing did affect the expression of those four plasma exosomal miRNAs at 3 mo of grazing.

Using the predicted target genes of these c-miRNAs, the GO terms and KEGG pathways associated with the downregulated c-miRNAs were analyzed by functional annotation analyses. Consequently, ‘Early endosome’ and ‘caveola’ were extracted in DAVID as terms categorized into the cellular component of the GO (Benjamini p<0.05). Moreover, KEGG pathways terms were also extracted as follows (Benjamini p<0.05): ‘Endocytosis’, pathways linked to carbohydrate and lipid metabolism and its endocrine regulation (‘Inositol phosphate pathway’, ‘Insulin signaling’, ‘Regulation of lipolysis in adipocyte’, ‘Adipocytokine signaling’), pathways related to environmental information processing (‘FoxO signaling’, ‘PI3K-Akt signaling’, ‘mTOR signaling’, ‘AMPK signaling’, etc.) (Table 3). Thus, the extracted GO terms and pathways indicate that the target genes of the downregulated c-miRNAs in the grazing cattle were associated with energy metabolism, the endocrine system, and environmental information processing.

### Alteration of lipid metabolism-related mRNA and miRNA expression in SCF and plasma NEFA during grazing

**Figure 3:**
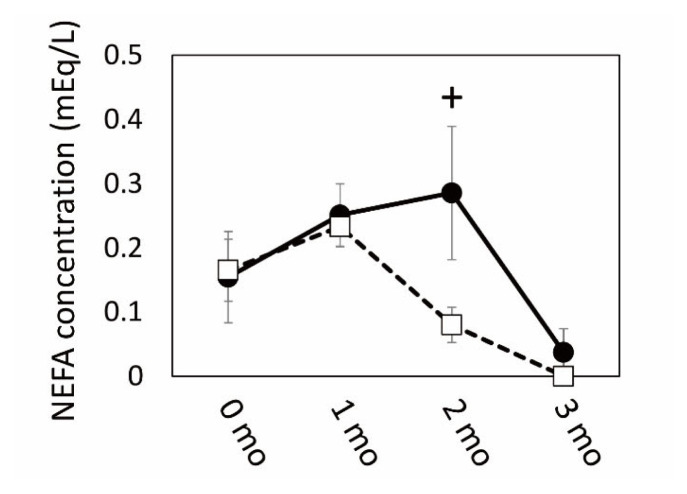
Changes in non-esterified fatty acid (NEFA) concentration during grazing period. Closed circles and open squares indicate grazing and housed in cattle, respectively. + indicates a difference between grazing and housed at p<0.10.

The results of microarray and GO analyses indicated that SCF was a highly responsive tissue in which numerous gene expression levels changed by grazing. These gene expression was associated with cellular and extracellular signaling, including cell-cell contact, and especially with the metabolism of carbohydrate and lipids, which might be affected by grazing as an environmental stimulus. Therefore, we next conducted a qPCR analysis of gene expression related to fatty acid and lipid metabolism in the SCF tissue of the two cattle groups. The plasma NEFA concentration increased in the grazing cattle until 2 mo of the grazing period had passed, followed by a subsequent decrease (Figure 3). Plasma NEFA appeared to be higher in the grazing cattle than in the housed cattle at 2 mo (p = 0.10).

**Figure 4:**
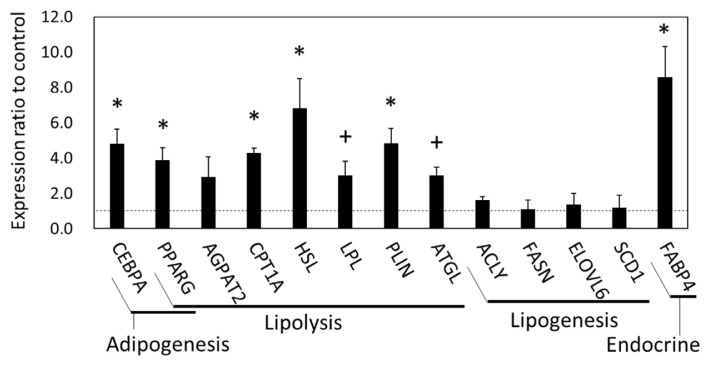
Gene expression related to adipogenesis and lipid metabolism in subcutaneous fat of grazing cattle. The ratios of the normalized gene expression of grazing cattle to housed cattle are shown in columns (level of housed cattle is indicated with dashed line). Ribosomal protein L7 (RPL7) was used as the internal control. Error bars indicate standard error. * and + indicate differences between grazing and housed cattle at p<0.05 and p<0.10, respectively.

In the qPCR results, the gene expression of CCAAT enhancer binding protein α (*CEBPA*), peroxisome proliferator-activated receptor γ (*PPARG*), hormone-sensitive lipase (*HSL*, encoded in *LIPE*), carnitine palmitoyltransferase 1A (*CPT1A*), perilipin 1 (*PLIN1*), *FABP4* (p<0.05), adipose triglyceride lipase (*ATGL*, also called *PNPLA2*) (p = 0.060), and lipoprotein lipase (*LPL*) (p = 0.073) in the SCF was higher in the grazing cattle than in the housed cattle (Figure 4). Meanwhile, the gene expression of 1-acylglycerol-3-phosphate O-acyltransferase 2 (*AGPAT2*), ATP citrate lyase (*ACLY*), ELOVL fatty acid elongase 6 (*ELOVL6*), fatty acid synthase (*FASN*), and stearoyl-coenzyme A desaturase 1 (*SCD1*) did not significantly differ between the two cattle groups (p>0.10). Thus, the expression of the lipolytic genes, but not that of the lipogenic genes was upregulated in the grazing cattle, as compared to the housed cattle.

**Figure 5:**
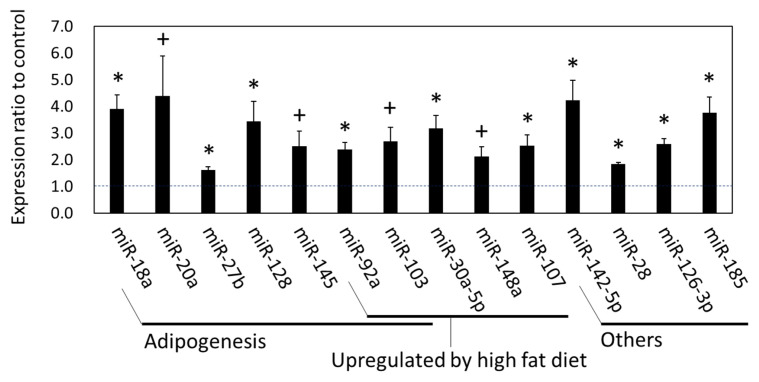
MicroRNA expression in subcutaneous fat of grazing cattle. The ratios of the normalized gene expression of grazing cattle to housed cattle are shown in columns (level of housed cattle is indicated with dashed line). RNA U6A small nuclear (RNU6) was used as the internal control. Error bars indicate standard error. * and + indicate differences between grazing and housed at p<0.05 and p<0.10, respectively.

On the other hand, the qPCR results also showed that miRNA expression in the SCF was altered by grazing. The expression of miR-18a, miR-27b, miR-28, miR-30a-5p, miR-92a, miR-107, miR-126-3p, miR-128, miR-142-5p, miR-185 (p<0.05), miR-20a (p = 0.087), miR-103 (p = 0.058), miR-145 (p = 0.066), and miR-148a (p = 0.073) in the SCF was higher in the grazing than in the housed group (Figure 5). In the GO analysis using the predicted targets of the upregulated SCF miRNAs, GO terms such as ‘3′-UTR-mediated mRNA destabilization’, ‘Multicellular organism growth’ (biological process; Benjamini p<0.05), ‘AMPK signaling pathway’, ‘Insulin signaling pathway’, ‘PI3K-Akt signaling pathway’, and ‘Regulation of lipolysis in adipocytes’ (KEGG pathway; Benjamini p<0.01) were extracted. These GO terms are in concordance with those resulting from the >1.5-fold changed mRNAs seen in the microarray analysis. Thus, the qPCR and subsequent bioinformatic results indicate that changes in miRNA expression in the SCF of grazing cattle were associated with lipid metabolism.

**Figure 6:**
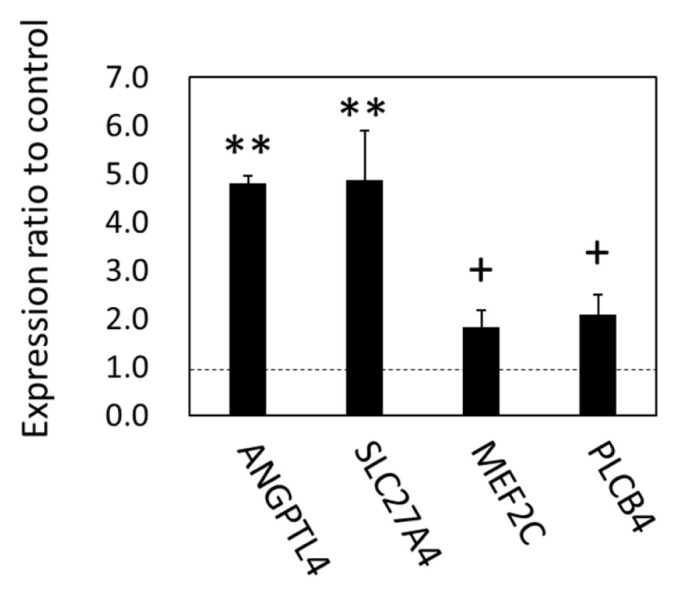
mRNA expression in the *biceps femoris* muscle of grazing cattle. The ratios of the normalized gene expression of grazing cattle to housed cattle are shown as columns (level of housed cattle is indicated with dashed line). β-Actin was used as the internal control. Error bars indicate standard error. ** and + indicate differences between grazing and housed at p<0.01 and p<0.10, respectively.

As the gene expression of lipolytic regulation and an organ crosstalk mediator, FABP4, were upregulated in SCF, we examined the expression of metabolic genes in the BFM by focusing on lipids and carbohydrate, in order to test the hypothesis that changes in SCF metabolism were linked to fatty acid metabolism-related gene expression in the skeletal muscle. As the results of qPCR showed, angiopoietin-like protein 4 (*ANGPTL4*), solute carrier family 27 member 4 (*SLC27A4*, encoding FATP4) (p<0.05), myocyte enhancer factor-2 (p = 0.071), and phospholipase C beta 4 (*PLCB4*; p = 0.055) were upregulated in BFM (Figure 6). The results indicated that the expression of genes in cattle related to long chain fatty acid (LCFA) uptake (*ANGPTL4* and *FATP4*), the response to LCFA, the stimulation of adipose lipolysis, and interorgan signaling (*ANGPTL4*) were all affected by grazing.

## DISCUSSION

### Grazing affected gene expression associated with exosomes prominently in SCF

In the present study, we observed the potential involvement of exosomes in energy homeostasis in the SCF, BFM, and liver during grazing of cattle. The results of mRNA and miRNA transcriptomics revealed that mRNA and miRNA transcriptomes in the SCF were highly responsive compared to those of the BFM and liver in the cattle from the grazing group. The following GO analyses of the >1.5-fold changed gene expression indicated that gene expression associated with exosome components was upregulated in the BFM, while it was downregulated in the SCF and liver tissues in the grazing cattle. This finding was further supported by the qPCR results showing the upregulation of genes associated with exosome generation or secretion (*CHMP4A*, *VAMP7*, *VPS26A*, *VPS37A*, *TSG101*) and exosome internalization (*MFGE8*, *RHOA*, *FLOT1*) [24,25] in the SCF; some of which were also upregulated in the BFM, albeit to a lesser extent than in the SCF. Considering that exosome component-related gene expression was downregulated in the SCF, exosome generation and internalization—but not secretion—might be active in the SCF of grazing cattle, which is suggestive of SCF crosstalk with BFM. Intriguingly, Thomou et al [26] demonstrated that adipose tissue is a major source of exosomal miRNAs known to play a role in glucose homeostasis, as demonstrated in a genetically engineered mouse model in which fat cells lacked Dicer, a critical miRNA-processing enzyme [26], suggesting important roles of adipose in regulation of circulating exosomal miRNA traffic.

The potential role of exosomes as a mediator in organ cross-talk was also indicated by the functional annotation of the potential c-miRNA target genes. In the grazing cattle of this study, circulating miR-142-5p and miR-21-5p levels significantly decreased (p<0.05), and miR-19b and miR-16a levels also showed a tendency toward decrease with grazing (p<0.10), as compared to the corresponding levels in non-grazing, housed cattle. A similar influence of grazing on miR-19b and miR-142-5p levels was also observed in our previous studies [4,6]. According to the functional annotation, the target genes of the downregulated c-miRNAs were associated with the endosome and caveola, which are thought to be essential for exosome generation and internalization [24,25], suggesting involvement of the c-miRNAs in exosome generation and uptake at the recipient organs. Thus, the results from transcriptomic and functional annotation analyses of those secretory organs suggest that grazing prompted communication between SCF and other bovine tissues utilizing the circulating exosome. The decrease in c-miRNA such as miR-142-5p might be due to the intake of miRNA-loading exosomes in the SCF, as suggested by the upregulation of gene expression levels of *MFGE8*, *RHOA*, *FLOT1*, *CD81*, and *CAV1* in the tissue.

Moreover, functional analysis yielded GO terms related to energy metabolism, the endocrine system, and environmental information processing in the SCF and BFM. One of the changed c-miRNAs, miR-142-5p, which was previously shown to be affected by grazing [6], is known to be involved in a variety of cell biological events in organogenesis and homeostasis [21] including fatty acid metabolism [27,28]. Given that exosomal c-miRNAs are employed in cell-cell communication, the present results suggest that energy metabolism and environmental information processing might be activated in potential c-miRNA recipient cells that participate in organ crosstalk. Our present results also suggest that such grazing-induced alteration of c-miRNA expression could be caused by altered exosome secretion and/or uptake activity in the SCF.

### Gene expression changes in SCF and BFM of grazing cattle associated with energy metabolism and interorgan signals

The transcriptomics and functional annotation analysis results revealed that gene expression levels associated with carbohydrate and lipid metabolism were affected in the BFM and SCF of grazing cattle. In the SCF, the expression of genes related to lipolysis (*CPT1A*, *HSL*, *LPL*, *PLIN*, *ATGL*, *FABP4*) was upregulated in grazing cattle. In contrast, lipogenic gene expression (*ACLY*, *FASN*, *ELOVL6*, *SCD1*) was not similarly affected in the grazing group, as demonstrated by the qPCR results. As shown by the elevated mRNA and/or protein levels of HSL and ATGL in the adipose tissue with exercise [29,30], the upregulation of gene expression levels related to lipolysis was dominantly enhanced by grazing in this study. These results revealed that metabolism in the SCF of grazing cattle was driven toward NEFA generation by triglyceride hydrolysis [31]. This finding is supported by the observed trend of upregulated expression of *PPARG*, which positively regulates *ATGL* mRNA and the protein expression in mature adipocytes [32]. Moreover, given that NEFA increased more in the plasma of the grazing cattle than in the housed cattle after 2 mo of grazing, it is likely that lipolytic products could have been released and contributed to the increase in circulating NEFA in grazing cattle. It is possible that the higher relative plasma NEFA concentration in the grazing cattle than in the housed cattle at 2 mo reflected greater NEFA-releasing activity of the SCF in the grazing cattle. Subsequent NEFA decreases in the grazing cattle suggest that circulating NEFA uptake by the skeletal muscles exceeded NEFA secretion by the SCF.

A secreted adipokine, *FABP4*, was also upregulated in the SCF of the grazing cattle. As FABP4 plays roles in maintaining adipocyte homeostasis, and in regulating lipolysis and adipogenesis through interaction with HSL and PPARG [33,34], it is suggested that the upregulation of *FABP4* expression enhances lipolysis and subsequent NEFA generation. Meanwhile, activation of energy metabolism via lipolysis in the SCF was suggested by the present functional annotation results, which was attributed to the alteration of carbohydrate and lipid metabolism-related gene expression. Collectively, these results revealed that energy metabolism in the SCF was activated by grazing to generate fatty acids as the energy substrate.

It is likely that during grazing, miRNA upregulation affected lipolysis in the SCF via target gene suppression. In the functional annotation analysis, a KEGG pathway term, i.e., ‘Regulation of lipolysis in adipocytes’, was extracted from the target genes of the upregulated miRNAs (Benjamini p<0.01). As mentioned above, this finding is in line with the transcriptomic analysis finding of upregulated gene expression related to lipolysis, which further indicates that miRNAs were involved in the upregulation of lipolytic gene expression and the potential changes in lipid metabolism in the SCF of grazing cattle.

**Figure 7:**
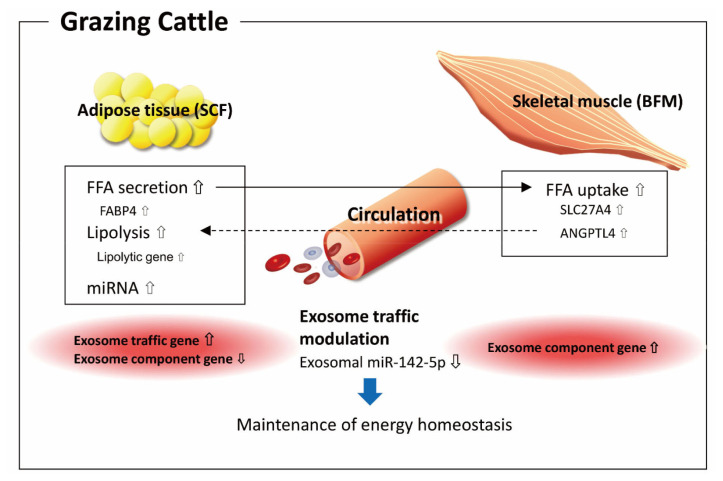
Hypothesized adipose-skeletal muscle organ crosstalk for energy homeostasis in grazing cattle. Lipolysis in subcutaneous fat (SCF) induced by grazing stimulation could generate non-esterified fatty acid (NEFA) and contribute to increases in circulating NEFA, which could then result in NEFA uptake by the *biceps femoris* muscle (BFM), with NEFA serving as an energy substrate in cattle. Exosome traffic might be involved in SCF-BFM organ crosstalk regarding energy homeostasis.

These changes in gene expression associated with energy metabolism could be linked to adipose-skeletal muscle axis organ crosstalk in grazing cattle. Similarly to human lipolysis, lipolysis in the SCF of cattle—induced by the stimulus of grazing in the latter case—could generate NEFA and contribute to increases in circulating NEFA, which may in turn result in NEFA uptake as an energy substrate in the BFM (Figure 7) [7]. The expression of a fatty acid transporter, *SLC27A4* (FATP4) protein, is enhanced by endurance exercise in human skeletal muscle to increase LCFA intake [35]. Moreover, we observed upregulated *ANGPTL4* expression in the BFM of grazing cattle. Production and secretion of ANGPTL4 from skeletal muscle is induced by LCFA [36]. In addition, as levels of ANGPTL4 have been shown to positively correlate with NEFA levels in the plasma and in adipose tissue lipolysis [36], the present results suggest that the grazing-induced *ANGPTL4* upregulation in the BFM could have been due to increases in plasma NEFA in the grazing cattle of this study. Furthermore, the C-terminal fibronectin-like domain (FLD) of ANGPTL4 stimulates lipolysis in primary adipocytes, and the FLD induces adipose lipolysis when increased in the circulation in mice [37], suggesting that the present *ANGPTL4* upregulation in the BFM provided feedback to the SCF to accelerate the lipolysis. Thus, the upregulation of *SLC27A4* and *ANGPTL4* observed in the BFM of grazing cattle suggests that the BFM takes part in energy homeostasis via organ crosstalk with the SCF in grazing cattle.

On the other hand, it has been shown that exosomes secreted from adipose tissue and skeletal muscle are incorporated into various tissues and can modulate gene expression related to glucose homeostasis *in vivo* [26]. As indicated by the results of the present functional annotation, it is likely that both the SCF and the BFM utilize circulating exosomes to participate in organ crosstalk for the maintenance of energy homeostasis in grazing cattle (Figure 7). In this study, a decrease in exosomal miR-142-5p in the plasma of the grazing cattle might have been involved in organ crosstalk for the maintenance of energy metabolism, as many target genes of miR-142-5p are associated with homeostasis. The decrease in circulating miR-142-5p could thus be associated with the observed increases in miR-142-5p in SCF.

In this study, we used four steers, which is a minimal number for experiments. Nevertheless, our finding of significant changes in transcriptome changes agreed with our previous results of miR-19b and miR-142-5p in grazing cattle [4,6] as mentioned above, as well as those of our previous gene expression and histochemical results (unpublished). This suggests that the overall tendency of transcriptional changes observed in the steers would be reproducible and not contradict what was observed in studies using larger numbers of animals, although further analyses are necessary to confirm those results.

## CONCLUSION

In grazing JSH cattle, mRNA and miRNA transcriptomes in the SCF largely changed in association with locomotive activity. The present transcriptomic results revealed that grazing induced changes not only in circulating exosomes, but also in energy metabolism, suggesting fatty acid generation in the SCF under regulation by miRNAs. Transcriptomic changes in exosome and energy metabolism were also observed in the BFM, indicating that the SCF and BFM participated in balancing circulating exosomes and energy homeostasis with fatty acid regulation in grazing cattle. Changes in c-miRNA expression could be linked to altered exosome secretion and/or intake activity in the SCF.

## Figures and Tables

**Table 1:** Genes with >1.5 fold-changed expression in subcutaneous fat, *biceps femoris* muscle, and liver tissues of grazing cattle in microarray analysis

Expression	Changes	SCF	BFM	Liver
mRNA	Increase	2,079 (169)	839 (152)	1,653 (112)
	Decrease	2,086 (249)	2,940 (133)	1,040 (106)
microRNA	Increase	46 (15)	10 (3)	7 (1)
	Decrease	10 (2)	2 (1)	0

SCF, subcutaneous fat; BFM, *biceps femoris* muscle.

The numbers of genes with >1.5-fold changed expression are shown. The numbers in parentheses are those with >2.0-fold changes.

**Table 2:** Gene ontology terms extracted from genes with >1.5-fold changed expression in subcutaneous fat, *biceps femoris* muscle, and liver of grazing steers

Gene expression	Category	Parent terms	Term	Fold enrichment	Benjamini
SCF					
Up	Cellular component	Extracellular region	GO:0005615~extracellular space	1.441	0.000682028
		Intrinsic component of membrane	GO:0005887~integral component of plasma membrane	1.443	0.002449399
			GO:0031225~anchored component of membrane	3.493	0.030828595
	KEGG pathway	Cellular community (Cellular process)	bta04530:Tight junction	2.609	0.002015041
		Endocrine system	bta04922:Glucagon signaling pathway	2.182	0.036999044
			bta03320:PPAR signaling pathway	2.397	0.040671086
		Signaling molecules and interaction (Environmental Information Processing)	bta04080:Neuroactive ligand-receptor interaction	1.690	0.008991485
Down	Cellular component	Extracellular region	GO:0005615~extracellular space	1.587	2.1235E-08
			GO:0005578~proteinaceous extracellular matrix	2.483	3.68628E-08
		Extracellular vesicle	GO:0070062~extracellular exosome	1.541	0.006696852
	KEGG Pathway	Carbohydrate metabolism	bta00650:Butanoate metabolism	3.082	0.035233296
		Cell-substrate adherens junction	bta04510:Focal adhesion	1.784	0.00759504
		Circulatory system	bta04270:Vascular smooth muscle contraction	1.885	0.025878831
		Development	bta04360:Axon guidance	1.933	0.016585302
		Immune system	bta04660:T cell receptor signaling pathway	1.890	0.037777158
		Lipid metabolism	bta00072:Synthesis and degradation of ketone bodies	4.706	0.047458464
		Metabolism	bta01100:Metabolic pathways	1.209	0.034155622
		Metabolism of other amino acids	bta00480:Glutathione metabolism	2.876	0.00525438
		Signaling molecules and interaction (Environmental Information Processing)	bta04512:ECM-receptor interaction	2.777	0.000241657
			bta04514:Cell adhesion molecules (CAMs)	2.129	0.001290428
			bta04151:PI3K-Akt signaling pathway	1.567	0.009244276
			bta04015:Rap1 signaling pathway	1.653	0.020605553
			bta04020:Calcium signaling pathway	1.598	0.049202032
BFM					
Up	Cellular component	Cell-substrate adherens junction	GO:0005925~focal adhesion	2.888	4.56791E-07
		Collagen-containing extracellular matrix	GO:0005604~basement membrane	4.187	3.57E-02
		Contractile actin filament bundle	GO:0001725~stress fiber	5.107	3.41E-03
		Extracellular region	GO:0031012~extracellular matrix	2.761	4.15E-02
			GO:0005615~extracellular space	1.933	9.21753E-07
			GO:0005578~proteinaceous extracellular matrix	3.102	9.66E-05
		Extracellular vesicle	GO:0070062~extracellular exosome	1.551	1.14E-06
		Plasma membrane raft	GO:0005901~caveola	4.271	4.56E-02
	KEGG pathway	Amino acid metabolism	bta00280:Valine, leucine and isoleucine degradation	4.183	1.45E-02
		Carbohydrate metabolism	bta00640:Propanoate metabolism	6.435	8.13E-03
		Cellular community (Cellular process)	bta04510:Focal adhesion	2.413	8.61E-03
		Circulatory system	bta04270:Vascular smooth muscle contraction	2.812	1.59E-02
		Endocrine system	bta03320:PPAR signaling pathway	4.780	9.46E-05
		Lipid metabolism	bta00071:Fatty acid degradation	6.631	6.69E-05
			bta01212:Fatty acid metabolism	5.664	1.45E-04
		Signaling molecules and interaction	bta04512:ECM-receptor interaction	3.365	9.39E-03
		Transport and catabolism	bta04145:Phagosome	2.383	3.42E-02
Down	KEGG pathway	Carbohydrate metabolism	bta00562:Inositol phosphate metabolism	2.830	4.88E-05
		Cellular community (Cellular process)	bta04550:Signaling pathways regulating pluripotency of stem cells	1.859	8.18E-03
		Endocrine system	bta04920:Adipocytokine signaling pathway	2.022	3.56E-02
		Immune system	bta04064:NF-kappa B signaling pathway	2.760	5.02E-06
			bta04650:Natural killer cell mediated cytotoxicity	1.963	7.72E-03
			bta04062:Chemokine signaling pathway	1.591	3.21E-02
			bta04621:NOD-like receptor signaling pathway	2.252	3.71E-02
			bta04620:Toll-like receptor signaling pathway	1.777	4.70E-02
		Signal transduction (Environmental Information Processing)	bta04015:Rap1 signaling pathway	1.912	1.07E-04
			bta04014:Ras signaling pathway	1.802	3.90E-04
			bta04010:MAPK signaling pathway	1.745	5.21E-04
			bta04070:Phosphatidylinositol signaling system	2.197	1.64E-03
			bta04151:PI3K-Akt signaling pathway	1.427	2.83E-02
		Signaling molecules and interaction (Environmental Information Processing)	bta04514:Cell adhesion molecules (CAMs)	1.724	1.94E-02
Liver					
Up	KEGG pathway	Cell growth and death	bta04110:Cell cycle	2.292	0.044756741
Down	Cellular component	Cell-substrate adherens junction	GO:0005925~focal adhesion	1.893	0.035663533
		Collagen-containing extracellular matrix	GO:0005604~basement membrane	4.057	0.019501063
		Extracellular region	GO:0005615~extracellular space	1.564	0.005789821
			GO:0005578~proteinaceous extracellular matrix	2.312	0.030412659
		Extracellular vesicle	GO:0070062~extracellular exosome	1.798	0.017504108
		Intrinsic component of membrane	GO:0005887~integral component of plasma membrane	1.674	0.003288116
		Protein-containing complex	GO:0005581~collagen trimer	4.290	0.004766008

SCF, subcutaneous fat; BFM, *biceps femoris* muscle; KEGG, Kyoto encyclopedia of genes and genomes.

**Table 3:** Gene ontology terms extracted from predicted target genes of altered c-miRNAs in grazing cattle

Category	Parent terms	Term	Fold enrichment	Benjamini
Cellular component				
	Protein-containing complex	GO:0043235~receptor complex	2.21802	0.002589508
		GO:0017053~transcriptional repressor complex	2.57576	0.014399863
	Endomembrane system	GO:0005794~Golgi apparatus	1.43911	0.002829639
		GO:0005802~trans-Golgi network	2.02722	0.005051763
	Intracellular part	GO:0000151~ubiquitin ligase complex	2.42797	0.005023967
	Endosome	GO:0005769~early endosome	1.83983	0.008947192
	RNAi effector complex	GO:0016442~RISC complex	4.68320	0.022571292
	Plasma membrane raft	GO:0005901~caveola	2.43266	0.037816814
KEGG pathway				
	Transport and catabolism	bta04144:Endocytosis	2.27855	3.93E-11
	Cellular community	bta04510:Focal adhesion	2.27855	7.08E-09
	Environmental information processing	bta04010:MAPK signaling pathway	2.10344	1.47E-08
		bta04068:FoxO signaling pathway	2.58063	3.71E-08
		bta04015:Rap1 signaling pathway	2.18006	4.58E-08
		bta04014:Ras signaling pathway	2.01676	7.50E-07
		bta04310:Wnt signaling pathway	2.27023	5.64E-06
		bta04151:PI3K-Akt signaling pathway	1.72861	7.70E-06
		bta04350:TGF-beta signaling pathway	2.49816	6.58E-05
		bta04071:Sphingolipid signaling pathway	2.15987	1.26E-04
		bta04150:mTOR signaling pathway	2.76129	1.33E-04
		bta04070:Phosphatidylinositol signaling system	2.11579	0.001117233
		bta04066:HIF-1 signaling pathway	2.09442	0.001309344
		bta04152:AMPK signaling pathway	1.78961	0.010445935
	Cell motility	bta04810:Regulation of actin cytoskeleton	2.08599	6.44E-07
	Endocrine system	bta04910:Insulin signaling pathway	2.28692	5.18E-06
		bta04923:Regulation of lipolysis in adipocytes	2.15426	0.021211677
		bta04920:Adipocytokine signaling pathway	1.98169	0.021678116
	Folding, sorting and degradation	bta04120:Ubiquitin mediated proteolysis	2.23247	1.15E-05
	Carbohydrate metabolism	bta00562:Inositol phosphate metabolism	1.87739	0.042685514

KEGG, Kyoto encyclopedia of genes and genomes.
