# Supplementary material for: Coordinated alteration of mRNA-microRNA transcriptomes associated with exosomes and fatty acid metabolism in adipose tissue and skeletal muscle in grazing cattle
Source: Asian-Australas J Anim Sci. 2019 Dec 24;33(11):1824–36. doi: 10.5713/ajas.19.0682 (PMC7649083; doi:10.5713/ajas.19.0682)
Supplement: Supplementary file 1 [file ajas-19-0682-suppl.pdf]

Table 1. Primer sequence and design used in this study

Target	Direction	Sequence	Accession
RPL7	fw	GTCATCAGGATCAGAGGTATCAATGGTGTG	NM_001113217.1
	rv	AAGGGCCACAGGAAGTTGTTTGCTTCTTTG	NM_001113217.1
ACLY	fw	CTGCCATGCTCCAAGGAAAGAG	NM_001037457.1
	rv	TTGTGACCCCAGTAAAACTTCTGT	NM_001037457.1
AGPAT2	fw	CTGTGATTTACTCCAGCTTCTCCT	NM_001080264.1
	rv	TCTTGGATATGTGGAAGAAGTGCG	NM_001080264.1
ATGL	fw	CTTCCTCGGCGTCTACCATATC	FJ798978.1
	rv	CCTTGGACACCTCGATGATGTTG	FJ798978.1
CAV1	fw	AACCTAGTTTTCCCAAACGCAG	XM_005205391.3
	rv	CTTGCTTCTCGTTCATTTCCTCTG	XM_005205391.3
CEBPA	fw	GTAAGCCAGGACCAGGAGATTC	BC149006.1
	rv	TCCTTTAATACTAGAGTTGCCGGG	BC149006.1
CHMP4	fw	GGAAGAAAAGGTTGGAACAGCAG	NM_001128504.2
	rv	AGTTCATCCACCTTGTCAATGTCC	NM_001128504.2
CPT1A	fw	CCTGCTTCCCTCTGCTCTTT	NM_001304989.1
	rv	GTCGCCATTGACGAATTCAGG	NM_001304989.1
ELOVL6	fw	AGCACCCGAACTAGGAGATACAAT	BC148954.1
	rv	TACCAGGAGTACAGAAGCACAGTGA	BC148954.1
FABP4	fw	CTCCAGATGACAGGAAAGTCAAGA	NM_174314.2
	rv	ATGCTCTCTCATAAACTCTGGTGG	NM_174314.2
FASN	fw	CCTCAAGATGAAGGTGGTGGAG	XM_005220997.2
	rv	GGCCCTGGGTTATATCGAGCTG	XM_005220997.2
FLOT1	fw	CATCTGCTAGACTCCCAGGTCC	NM_001076887.1
	rv	AACTCTTTCCCTTTCCAGTCAGTC	NM_001076887.1
HSL	fw	GATATCTGAAGAGGCCTGGGAGG	NM_001080220.1
	rv	GAAGAAGGCCATGTTGTCCTCTG	NM_001080220.1
LPL	fw	TCAGATGCCTTACAAAGTCTTCCA	NM_001075120.1
	rv	CACCTCCGTGTAAAGTAGAAAGGA	NM_001075120.1
PGC1A	fw	ATGAAACTGACTTCGAGCTGTACT	XM_015471550.1
	rv	CCAGAGAGTCATACTTGCTCTTGA	XM_015471550.1
PLIN1	fw	ACCTGGTTGGTTTCTGAGAAGTAA	NM_001083699.1
	rv	TAGGTCTTCTGGAAGCACTCAC	NM_001083699.1
PPARG2	fw	TATTCTCAGTGGAGACCGCC	NM_181024.2
	rv	CTGCACGTGTTCTGTCACAAT	NM_181024.2
RHOA	fw	GGAACAAGAAGGATCTTCGAAACG	NM_176645.3
	rv	CATCTTTGGTCTTTGCTGAACACT	NM_176645.3
RPL7	fw	GTCATCAGGATCAGAGGTATCAATGGTGTG	NM_001113217.1
	rv	AAGGGCCACAGGAAGTTGTTTGCTTCTTTG	NM_001113217.1
SCD1	fw	ACCTGGCTGGTGAATAGTGC	NM_173959.4
	rv	AAGTTGATGTGCCAGCGGTA	NM_173959.4
TSG101	fw	CACCAAATACTTCCTACATGCCAG	NM_001097995.2
	rv	CTCACTGATTGTGCCATCTCTACT	NM_001097995.2
VAMP7	fw	TCTTTGTCCTGTCGACCTGTTTAT	NM_001076302.1

	rv	TAAGACAGGTGGTTTCTACCTTGG	NM_001076302.1
VPS4B	fw	CTACCAACCTGCAGAAAGCCATA	NM_001076156.1
	rv	TGGCTTTATCACCTTGTGCTTCAT	NM_001076156.1
VPS26A	fw	AGGATATGACCCAACTCCAACAAT	NM_001075455.1
	rv	TGATTCTGGTGATTCAAATCGCTG	NM_001075455.1
VPS37A	fw	GGCAAGATTAAAAGTAGCTGCACA	NM_001046161.2
	rv	GTTGAAGTTTCTCTTCCTTGGCTC	NM_001046161.2

fw: forward primer, rv: reverse primer.
